# Deep learning-based classification of blue light cystoscopy imaging during transurethral resection of bladder tumors

**DOI:** 10.1038/s41598-021-91081-x

**Published:** 2021-06-02

**Authors:** Nairveen Ali, Christian Bolenz, Tilman Todenhöfer, Arnulf Stenzel, Peer Deetmar, Martin Kriegmair, Thomas Knoll, Stefan Porubsky, Arndt Hartmann, Jürgen Popp, Maximilian C. Kriegmair, Thomas Bocklitz

**Affiliations:** 1 Institute of Physical Chemistry and Abbe Center of Photonics (IPC), Friedrich-Schiller-University, Jena, Germany; 2 Leibniz Institute of Photonic Technology (IPHT), Jena, Germany; 3 Department of Urology, University of Ulm, Ulm, Germany; 4 Department of Urology, University Hospital Tübingen, Tübingen, Germany; 5 Pathology Munich-Nord, Munich, Germany; 6 Urological Hospital Munich-Planegg, Munich, Germany; 7 Department of Urology, Hospital Sindelfingen-Böblingen, University of Tübingen, Sindelfingen, Germany; 8 Institute of Pathology, University Medical Center of the Johannes Gutenberg University Mainz, Mainz, Germany; 9 Institute of Pathology, University of Erlangen, Erlangen, Germany; 10 Department of Urology, University Medical Centre Mannheim, Mannheim, Germany

**Keywords:** Cancer, Urogenital diseases, Cancer

## Abstract

Bladder cancer is one of the top 10 frequently occurring cancers and leads to most cancer deaths worldwide. Recently, blue light (BL) cystoscopy-based photodynamic diagnosis was introduced as a unique technology to enhance the detection of bladder cancer, particularly for the detection of flat and small lesions. Here, we aim to demonstrate a BL image-based artificial intelligence (AI) diagnostic platform using 216 BL images, that were acquired in four different urological departments and pathologically identified with respect to cancer malignancy, invasiveness, and grading. Thereafter, four pre-trained convolution neural networks were utilized to predict image malignancy, invasiveness, and grading. The results indicated that the classification sensitivity and specificity of malignant lesions are 95.77% and 87.84%, while the mean sensitivity and mean specificity of tumor invasiveness are 88% and 96.56%, respectively. This small multicenter clinical study clearly shows the potential of AI based classification of BL images allowing for better treatment decisions and potentially higher detection rates.

## Introduction

Bladder cancer is among the most common cancers and the leading cause of death in western countries^1^. The primary diagnosis and treatment of bladder cancer is based on endoscopic procedures. Here, the standard of health care is white light (WL) cystoscopy, which offers an excellent sensitivity and specificity to detect papillary tumors, but it misses a significant fraction of small and flat lesions^2,3^. To increase the detection rate of these lesions, modern imaging technologies such as photodynamic diagnosis (PDD) are highly recommended. According to a couple of meta-analyses, 40% of flat cancerous lesions are only detected in BL cystoscopy^4,5^. Consequently, the implementation of PDD can result in a change of the respective bladder cancer risk classification, and thus a more accurate therapy^6^. However, PDD harbors some significant drawbacks concerning its low specificity which ranges from 35 to 60%^4,5^. For instance, it is difficult to distinguish flat cancerous lesions from inflammable alterations following transurethral resection or instillation^6^. Moreover, the interpretation of PDD findings is highly subjective and may vary between observers. This accounts especially for less experienced endoscopists, where the rate of false positives is particularly high^7^. Finally, PDD supports the distinction of malignant and benign tissues but does not offer diagnostic information regarding tumor stage and grading.

Currently, brain-inspired deep neural networks (DNNs) have been revolutionizing artificial intelligence, and they have shown their potential for computer-aided diagnostic systems in various fields such as radiology^8^, histopathology^9^ and computational neuroscience^10^. In terms of image processing, deep neural networks (DNNs) exhibit the best performing models for object recognition and yield human performance levels for object categorization^11^. Typically, these DNNs mimic the mechanism of human brains by letting DNNs learn specific image features that improve the identification performance on new unlabeled data sets. In the basic architecture of a DNN, the neural network is trained by passing a data set of labeled images through multiple layers that consist of simple units called neurons. These neurons compute different linear combinations of specific image features captured from the labeled data set and pass the results into the next layer through a static nonlinearity, e.g., replacing negative values by zeros. The previous nonlinear layer is usually known as activation layer, and it is followed by pooling layers that aim to reduce the spatial dimension of the image features. Then, DNNs process the images as a sequence of visual representations in which each neuron detects a specific local region of the feature map in the previous layer while similar feature detectors exist across locations in the feature map^10^. Nonetheless, the term “Deep” in deep neural networks indicates that multiple layers of neurons are utilized in DNNs and improve their identification performance. Such training procedures are usually time consuming and require a large sample size of labeled images, which is rarely available for biomedical applications. Therefore, the concept of transfer learning of DNNs was introduced to deal with classification tasks on small data set. Thereby, the identification knowledge gained via training DNNs on a large annotated data set can be transferred to solve another classification task based on a new and small data set^12,13^. These strategies have shown a great potential for diagnostic classifications of biomedical images using relatively small sample sizes^14–17^. Further, implementing such deep learning models in biomedicine may increase sensitivity and specificity of diagnostic procedures and reduce inter-observer variance^18,19^. However, respective solutions in endoscopy are rare. Recently, Shkolyar and colleagues introduced a deep learning automated image processing platform for cystoscopy. The software was able to identify papillary lesions in videos from WL cystoscopy with a high sensitivity and specificity^20^. Similarly, a recent study established a classification system based on 233 images of bladder wall lesions that was able to identify cancerous formations with a very high sensitivity, but with a low specificity of 50%^21^. Although these preliminary findings are promising, further developments in automated image processing are highly appreciated in urological endoscopy. In this context, PDD was considered as an effective modern imaging technique that offers characteristic information about tumor morphology. This technology utilizes the fluorescence properties of an extrinsic metabolic substrate, which is differently metabolized in cancerous and healthy tissues^22^. Consequently, PDD images contain more comprehensive information as compared to WL images.

The aim of this study is to test the classification of a small BL image data set consisting of bladder tumor and healthy urothelium images. This test was accomplished using an automated image processing pipelines and deep convolutional neural networks (CNNs) as a first step to implement computer-aided diagnosis in urological endoscopy. Our workflow started by preprocessing the BL images to include regions containing bladder tissue only. Then, the identification performance of different pre-trained CNNs in predicting bladder cancer malignancy, invasiveness and grading was investigated via a fine-tuning-based transfer learning strategy. Shortly, a comparison between the implemented CNN models and bladder cancer ratings of two experienced urologists was performed on the basis of the classification sensitivity.

## Results

We present in this section the classification results of BL images using the previously explained fine-tuned CNNs. Overall, images from 216 different lesions were included and three classification tasks based on these BL images were established. In Table 1, the pathological results of the biopsied lesions are shown. We can see that the numbers of collected images per class are different; thus, class weights were considered to correct the unbalance within the data set while the CNNs were trained.

**Table 1:** Distribution of pathological staging after TUR-BT of the respective PDD positive lesions.

Histology	n (%)
Benign	74 (34.26%)
CIS	17 (7.87%)
Ta, LG	72 (33.33%)
Ta, HG	28 (10.18%)
T1, LG	1 (0.46%)
T1, HG	13 (06.02%)
≥ T2, any grade	11 (05.09%)
Low-grade	73 (33.80%)
High-grade	69 (31.94%)
Malignant	142 (65.74%)

The separation into low-grad and high-grade was made according to the WHO 2004 classification and malignant was defined as all samples diagnosed with any kind of bladder cancer.

### Identification of malignant bladder tumors lesions

**Figure 1:**
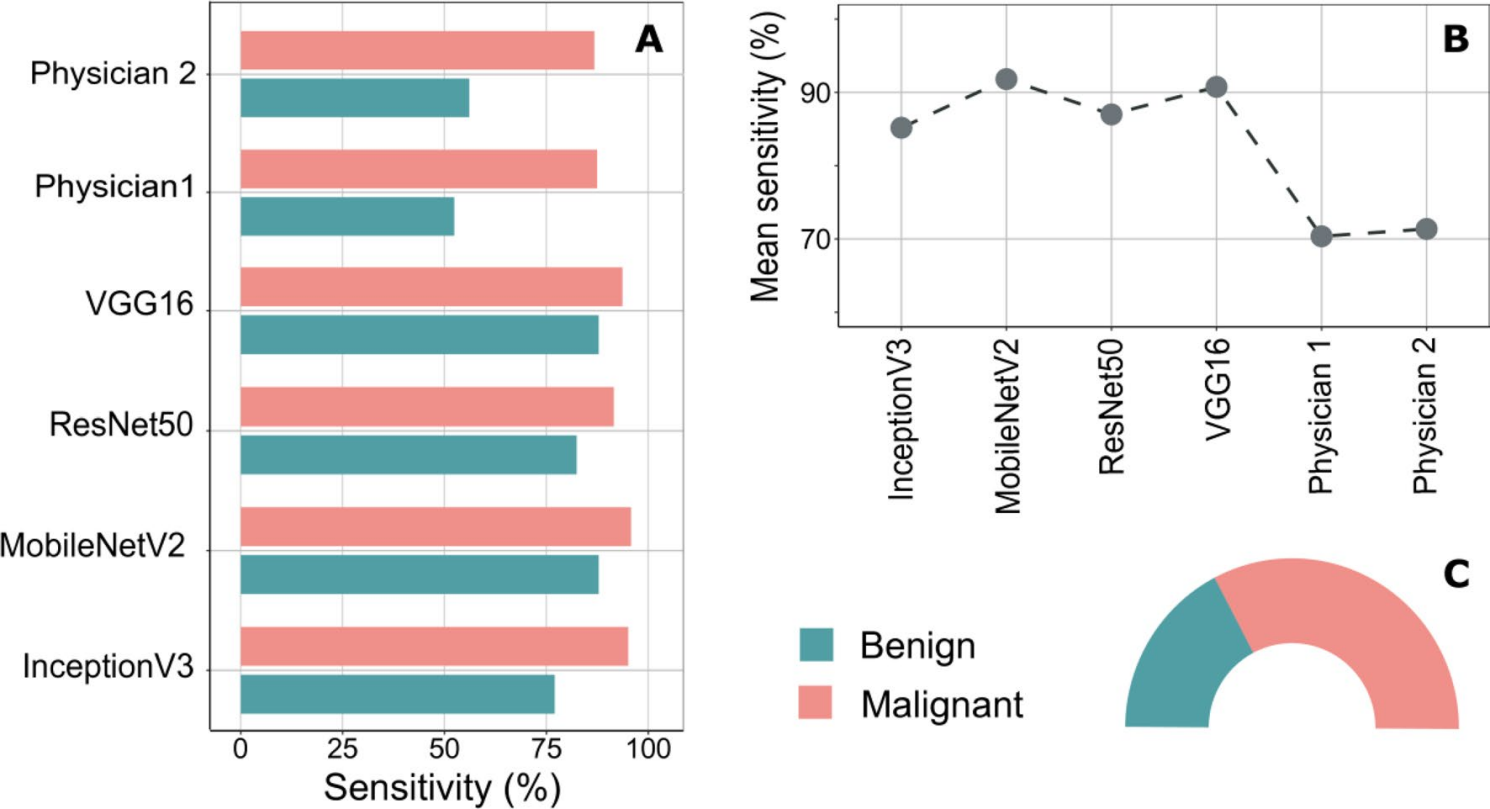
The identification results of the malignant tumor lesions in bladder. (**A**) The classification sensitivities of benign and malignant images based on the considered fine-tuned CNNs and the physician ratings. All CNNs could predict the malignant images quit well with sensitivity of at least 91% and specificity larger than 77%. (**B**) Comparison between the mean sensitivities of the fine-tuned CNNs and the physician ratings. The MobileNetV2 network followed by VGG16 network showed the best classification results with a mean sensitivity of 91.81% and 90.75%; respectively. (**C**) The class distribution of the BL image data set with respect to the percentage of malignant and benign images in the data set. Clearly, the number of malignant images is much larger than the number of the images collected from benign legions.

The goal of this task is to evaluate the performance of the fine-tuned CNNs in identifying malignant lesions within the BL images, which were collected in multiple centers. Therein, the prediction results obtained by the proposed CNNs were compared with the physician ratings and summarized in Table S1 and Fig. 1. In Fig. 1A, the classification sensitivities of CNNs based on the leave-10-patients-out cross-validation (L10PO-CV) and the physician ratings were visualized as bar charts. For this binary task, the highest classification sensitivity for malignant lesions and for benign lesions are 95.77% and 87.84%; respectively. Here, the fine-tuned MobileNetV2 network provided the best identification of malignant lesions while both fine-tuned MobileNetV2 network and fine-tuned VGG16 network introduced the highest sensitivity for the prediction of benign images. Moving to Fig. 1B, the mean sensitivities of all fine-tuned CNNs and both physicians are visualized. Clearly, the fine-tuned MobileNetV2 network features the highest mean sensitivity with the value of 91.81% followed by the fine-tuned VGG16 network with a mean sensitivity of 90.75%. It is also obvious that the performance of any fine-tuned CNN is at least 15% superior in their classification mean sensitivity as compared to the mean sensitivities obtained by both physician ratings. Nevertheless, the detailed confusion matrices of the pervious binary task are presented in Table S1 while the percentage of class sample sizes to all data set size is shown in Fig. 1C as pie chart.

### The identification of bladder cancer invasiveness (T-stage)

Because each stage of bladder cancer required a specific medical treatment, we were interested in comparing the classification results of the bladder cancer stages using the considered deep learning models with those obtained by the urologists. To do so, the acquired BL images were categorized into five classes representing benign tissue, carcinoma in situ (CIS) and the following three bladder cancer stages: Ta, T1 and ≥ T2. Table S2 shows the classification results of the bladder cancer invasiveness as a single confusion matrix reflecting all results of each model, i.e., results of CNNs and physician ratings.

**Figure 2:**
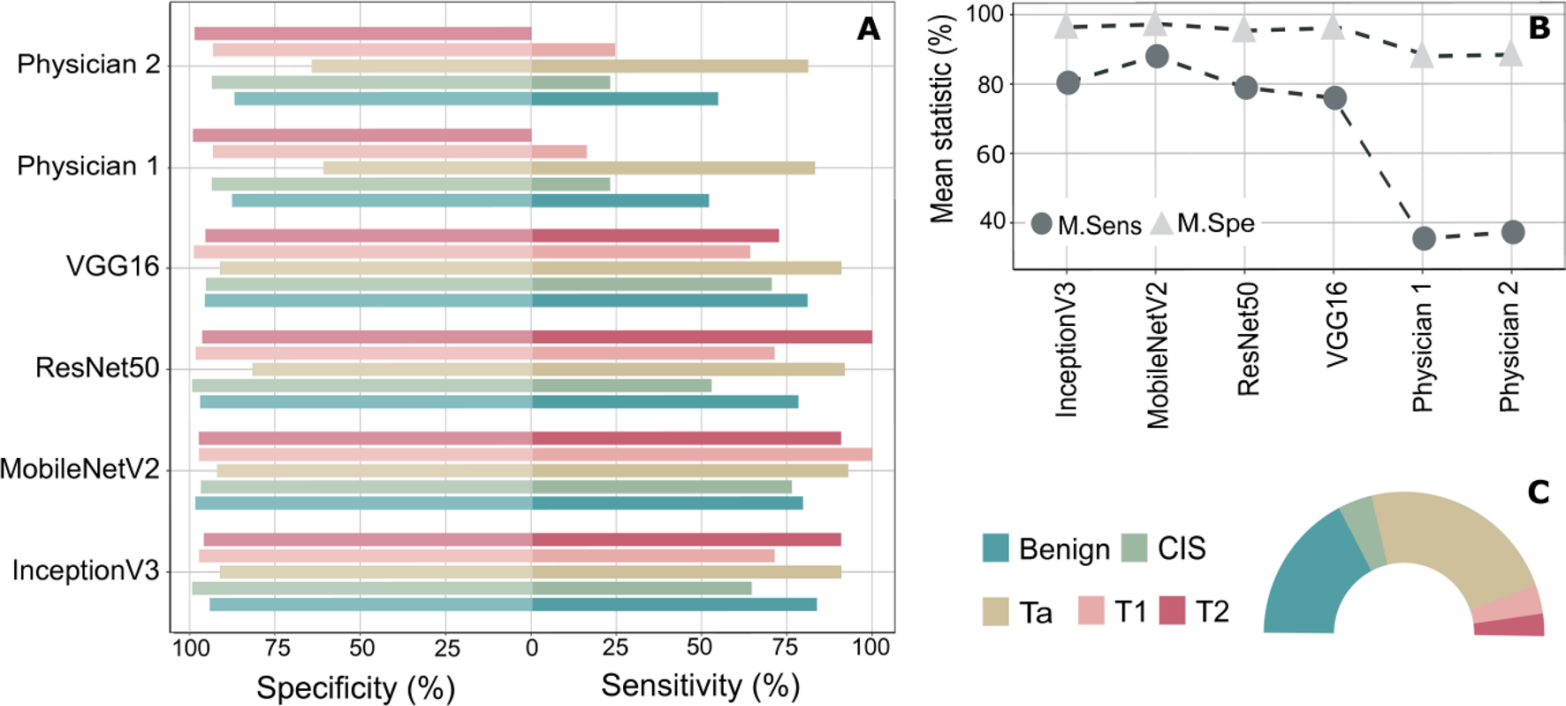
The identification of bladder tumor stage using the fine-tuned CNNs and urologist ratings. (**A**) The class sensitivities and specificities of the considered CNNs and both physician ratings as a two-directions bar chart. While both urologists could not assess well the last two invasive stages of bladder cancer, the detection of these tumor stages was quite good based on all CNNs (**B**) The obtained mean sensitivities and mean specificities for all classification models. The best classification results were achieved by the MobileNetV2 network with a mean sensitivity of 88% followed by the mean sensitivity obtained by the InceptionV3 network. The classification mean sensitivity decreased at least 50% when the same images were assessed by the urologists. (**C**) The class distribution of the BL image data set. The number of involved images for this task varies a lot from one class to another class.

In Fig. 2A, the class sensitivities and specificities of each model were plotted as a two-direction bar chart while in Fig. 2B the mean sensitivities and the mean specificities of the respective models were compared based on a point chart. Figure 2C visualizes the proportion of sample size for each class to the whole data set size as a pie chart. Clearly, the class sample sizes are quite different, and the sample size is quite small for the T1 and T2 cancer stages (≤ 15 images). Based on Fig. 2A, the best identification results of benign images were provided by the fine-tuned InceptionV3 network. Thereby, the observed sensitivity and specificity of benign images is 83.78% and 94.37%, respectively. Regarding image classification of CIS and the bladder cancer stage T1, the fine-tuned MobileNetV2 network introduced the best predictions with a class sensitivity of 76.47% for the CIS images and 100% for the T1 images. While the fine-tuned ResNet50 network presented the best identification of the T2 images with 100% classification sensitivity, the highest classification sensitivity for Ta images was obtained again using fine-tuned MobileNetV2 network. Here, the observed sensitivity of bladder cancer stage Ta is 93%. In contrast, both urologists misidentified almost all images of class CIS, T1 and T2, but they could assess the first stage of bladder cancer, i.e., Ta cancer stage, well. Overall, the highest mean sensitivity and the highest mean specificity were reached by the MobileNetV2 network based on the L10PO-CV as it is shown in Fig. 2B. The observed mean sensitivity and mean specificity of the previously mentioned CNN are 88% and 96.56%; respectively. However, the classification mean sensitivities dropped at least 50% when the BL images were accessed by any of both urologists.

### The differentiation of the bladder cancer grading

**Figure 3:**
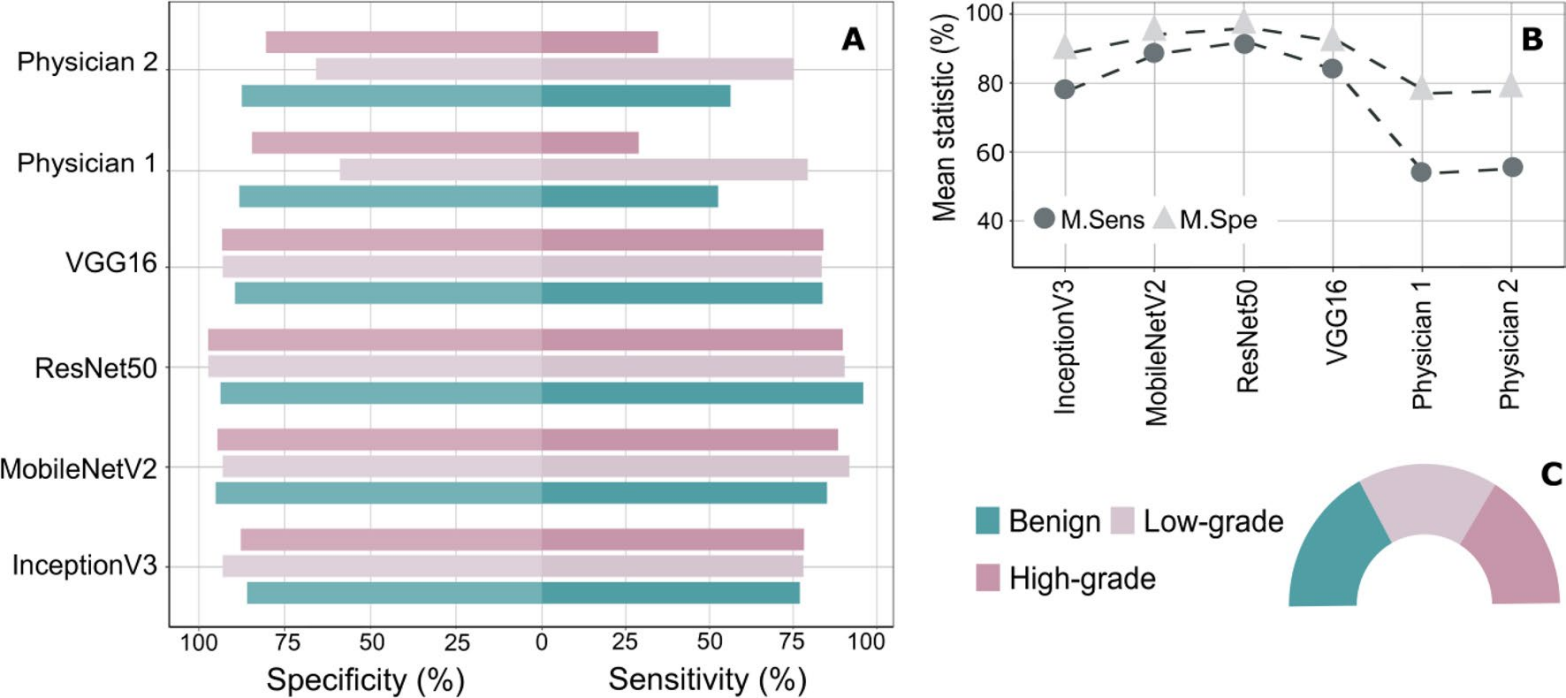
The classification results of bladder cancer grading. (**A**) Overview of the individual class sensitivity and specificity with respect to both physician ratings and the fine-tuned CNNs. (**B**) Summary plot of the mean sensitivities and mean specificities obtained by the fine-tuned CNNs and both physician’s ratings. An increase between 25 and 40% of the classification mean sensitivity can be observed if the fine-tuned CNNs were considered to identify bladder cancer grading based on the collected BL images. (**C**) Image class distribution with respect to the whole data size. Almost similar number of images were acquired from benign, low-grade, and high-grade lesions.

We present in this subsection the results of deep learning models and physician ratings in identifying the bladder cancer grading based on the BL image data set that was collected from a study involving multiple centers. In Table S3 and Fig. 3, the classification results obtained by the proposed fine-tuned CNNs and by the physician ratings are presented. Table S3 describes the detailed confusion matrices of all previous models and ratings.

The results showed that the fine-tuned ResNet50 network based on L10PO-CV is the best in predicting cancer grading followed by the fine-tuned MobileNetV2 network. Figure 3A depicts the sensitivities and specificities of all models as a two-directions bar chart while Fig. 3B shows a summary plot of the mean sensitivities and mean specificities of all previous models. We see in this figure that the ResNet50 network shows the highest-class sensitivity for high-grade cancer and benign images among the other models, i.e., CNNs and physician ratings. Thereby, the observed sensitivity of the high-grade images and the benign images has the values of 89.86% and 95.95%; respectively. For the identification of low-grade images, the MobileNetV2 network introduced the highest sensitivity in comparison to the other models. Here, the classification sensitivity of low-grade images using the MobileNetV2 network is 91.78%. The overall results indicate that the best performing model is the fine-tuned ResNet50 network while the lowest classification mean sensitivities were obtained by the urologist ratings. Therein, the mean sensitivity and mean specificity of the fine-tuned ResNet50 networks is 92.07% and 96.04%; respectively. On the other side, the mean sensitivity of the first and the second urologist is 53.71% and 55.17%, respectively.

## Discussion

In this contribution, we introduced the identification results of bladder cancer using BL endoscopic images acquired from four different urological departments. The data set consists of 216 BL images, that were recorded prior to resection of the respective lesions. The collected BL images were utilized to evaluate the ability of deep learning models in automating the classification of the endoscopic lesions and predicting histopathological results. The bladder cancer identification was demonstrated based on four deep CNNs, and the results were compared with those obtained by two experienced urologists. This comparison was evaluated to predict cancer malignancy, cancer invasiveness and cancer grading. For all these tasks, pre-trained versions of InceptionV3, MobileNetV2, ReNet50, and VGG16 networks were fine-tuned, and then they were evaluated using a L10PO-CV.

The results of the previous named CNNs showed that the fine-tuned MobileNetV2 network has the best performance in detecting images of malignant lesions with a sensitivity of 95.77% and a specificity of 87.84%. The detection performance of this MobileNetV2 network exceeds the performance of other imaging technologies typically used for enhancing bladder cancer detection. For example, probe-based techniques such as CLE or OCT provide sensitivity levels between 80 and 90% for the detection of malignant lesions^23,24^. This underlines the potential of automated image analysis systems in urological endoscopy. For such a classification task, i.e., malignancy identification, an increasing sensitivity should be the primary target. Therefore, the detection of all cancerous lesions during TUR-BT is important for correct identification of bladder cancer staging and adjuvant therapy stratification. Moreover, the failure to remove all tumor tissues is a main reason for high residual tumor rates in patients with intermediate and high-risk NMIBC requiring a 2nd TUR-BT^25^. Recently, an image analysis platform, named: CystoNet, was constructed and evaluated resulting in a sensitivity of 90.9% in detecting papillary bladder tumors^20^. However, unlike the fine-tuned MobileNetV2 network in this study, its specificity was low. Accordingly, Gosnell and colleagues introduced an endoscopy image-based classification system with high sensitivity, but it also suffered of the low specificity (~ 50%)^21^. Consequently, the image analysis technology used in this study has not only shown promising results regarding sensitivity, but also for specificity levels.

Moving to the classification of the cancer invasiveness (T stage), the fine-tuned MobileNetV2 network performed quite well for the presented multiclass task. Thereby, the classification sensitivity of each of the tumor stage T1 and T2 is 100% and 90.91% even though the image sample size per class was quite small (< 15 images per class). Overall, the mean sensitivity and the mean specificity of the fine-tuned MobileNetV2 based on the cross-validation is 88.02% and 96.56%; respectively. For the identification of bladder cancer grading, the fine-tuned ResNet50 network provided the best classification results compared to other CNNs. The observed mean sensitivity and mean specificity of the ResNet50 using the considered cross-validation strategy is 92.07% and 96.04%, respectively. However, the identification performances of both urologists were much worse (mean sensitivity between 35 and 37%) than the classification performance of any of the considered deep learning models.

Beside the challenges presented for identifying bladder cancer invasiveness and grading, flat malignant lesions constitute a challenging situation for urologists. Due to its flat growth pattern, the carcinoma in-situ (CIS) of the urinary bladder is hard to be detected in WL imaging^4^. In contrast to other organs, CIS of the urinary bladder has high-grade characteristics and is potentially invasive. Therefore, it is important to correctly identify and hence cure this cancer type. Although PDD can assist physicians and significantly increase the detection rate of CIS, such flat lesions remain hard to be characterized for urologists^5^. Furthermore, scar tissue and inflammation can mimic CIS characteristics; especially in BL cystoscopy resulting in a high number of false negative biopsies^26^. In this discourse, artificial intelligence-based cancer identification might be an effective tool for better classification of the respective urothelial lesions. Consequently, we were interested in testing the prediction quality of deep learning models in differentiating flat bladder lesions, that include images of benign and CIS lesions. This was achieved using the proposed fine-tuned CNNs on the collected images of the respective bladder lesions, e.g., CIS, and benign tissue. Similar hyperparameters and similar network architectures were used for the aforementioned CNNs with the L10PO-CV being the validation method. In Table S4, the results of the previous binary classification obtained by all considered CNNs were summarized with respect to the class sensitivities, then they were compared with the class sensitivities resulted from the classification of bladder tumor invasiveness, i.e., multiclass models. It turned out that the fine-tuned MobileNetV3 network based on the multiclass training performed the best in the differentiation between benign and CIS lesions with a mean sensitivity of 78.10%. Remarkably, the specificity of CIS lesions in the binary model was high reaching 90% using the InceptionV3 and ReNet50 networks. To improve and verify these findings, further clinical research is needed to enhance the low specificity, which introduces one of the major drawbacks of PDD imaging. Similar drawbacks exist in other imaging techniques such as Optical Coherence Tomography^27^. The advantage of improving the detection sensitivity and specificity of flat lesions would be of particular interest when PDD is utilized in an outpatient setting, where biopsies and resection are not possible^28^. Indeed, a recent study revealed that refuting suspicious lesions is one of the major motivations physicians to use adjunct imaging modalities^28^. Thus, cancer diagnosis based on the combination of imaging technologies and artificial intelligence would be highly appreciated in clinics. Nevertheless, our classification results of flat lesions were highly influenced by the sample size when the CNNs were considered to perform the binary classification, i.e., CIS vs. benign lesions. Therein, the fine-tuned CNNs were trained and validated on 91 BL images with unequal class sizes. However, we expect to achieve better classification performance if the previous mentioned challenges are addressed in future studies.

**Figure 4:**
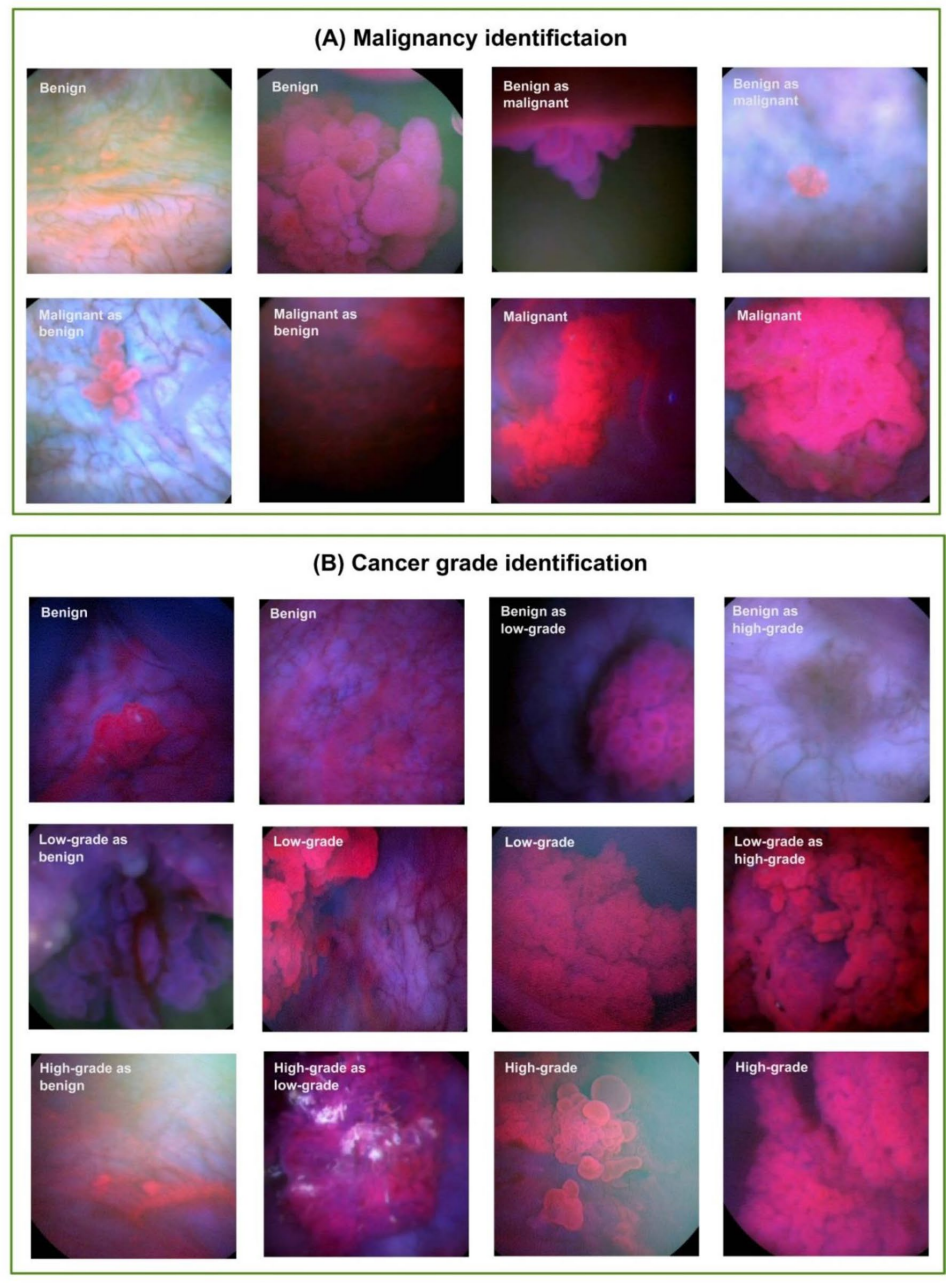
Examples of correctly predicted images and misclassified images using the fine-tuned CNNs.

In summary, transfer learning based on pre-trained CNNs enabled us to identify bladder cancer despite the small sample size and the unbalance in data set. For all tasks, the fine-tuned CNNs provided promising results. Moreover, the misclassification of BL images in most cases was expected due to the high variations between the images and due to other systematic errors. Figure 4 presents examples of correctly classified and misclassified images related to two of the considered classification tasks, i.e., the classification of malignant lesions and the classification of cancer grading. It is clear in this figure that the fluorescence of some images is very low while it is very spotty in others. Additionally, the urine fluorescence within some images may drown out the red fluorescence; therefore, these images were mostly incorrectly identified. Beside the fluorescence issues, some images depicted flat lesions while others were not close enough to capture the suspicious lesions. As a result, these images were also misclassified.

Nevertheless, the main limitations of the current study its retrospective design. Further, the image identification results provided by both urologists are not the typical procedure considered for such cancer diagnosis in clinical practice. Due to the low morbidity of additional biopsies, urologists tend to biopsy some to all suspicious lesions, which results in a high sensitivity and low specificity. Another limitation is the low number of BL image for all lesions, specifically for CIS lesions. These small sample sizes allowed for exploratory analyses on lesions. However, only BL images with obvious histopathological correlates were included while all four centers used equivalent clinical and technical set-ups.

## Conclusion

The results in this study demonstrated the potential of artificial intelligence-based classification models for the diagnostics of bladder cancer based on BL cystoscopic images. In this context, further studies need to be performed in order to build an automatic BL cystoscopic platform that assists physicians in identifying and classifying potential lesions of interest. Applying such platforms into clinical routines aims to assist surgeons and aids the cancer diagnoses. Potentially this technology could increase the detection rates of cancer and improve the relative low specificity of BL imaging. However, the system will not be considered to substitute the opinion of endo-urologists or pathologists for clinical decision making.

## Material and methods

### Image acquisition and pathological evaluation

A total of 216 BL images acquired during PDD-guided transurethral resection of bladder tumor (TUR-BT) were collected from four urological departments retrospectively and one image was taken for an individual patient. Therefore, every image represents a patient. Routine pathological evaluation was performed, and all tumors were classified according to the world health organization (WHO) classification 2004. Only endoscopic images recorded prior to the resection of the respective lesions were used while the distance from the endoscopy to the region of interest was not standardized. For PDD, intravesical instillation of 85 mg Hexaminolevulinathydrochlorid (Hexvix®, IPSEn Pharma, Boulogne, France) was performed 60 min prior to PDD. Imaging was performed using the Tricam II® system and a 30-degree Hopkins II optic (Karl Storz, Tuttlingen, Germany) in all centers. Further, two experienced urologists (CB and MCK, both > PDD 300 TUR-BTs) assessed the endoscopic images. Therein, the following distinctions were requested from the urologists and subsequently performed by different deep CNNs based on the PDD images only:

(i) Malignancy: malignant vs benign lesions
(ii) Tumor invasiveness (T-Stage): benign lesions vs carcinoma in situ (CIS) vs Ta vs T1 vs ≥ T2
(iii) Tumor grading: benign vs high-grade vs low-grade cancer
(iv) Flat lesions: benign vs CIS

For the previous classification tasks, the number of collected image per class is not equal; therefore, class weights were considered to correct this unequal class sizes within the data set while the classification models were trained.

Nevertheless, the data was collected retrospectively. Written informed consent was obtained, if possible. Data was analyzed and forwarded anonymized from the respective clinical center to all other study participants. This study was approved by the local ethical committee of the leading study side Mannheim (Ethics Committee II of the University of Heidelberg at the Medical Faculty Mannheim, Ref Number: 2015 549N MA) and in accordance with the Declaration of Helsinki.

### Image region of interest

**Figure 5:**
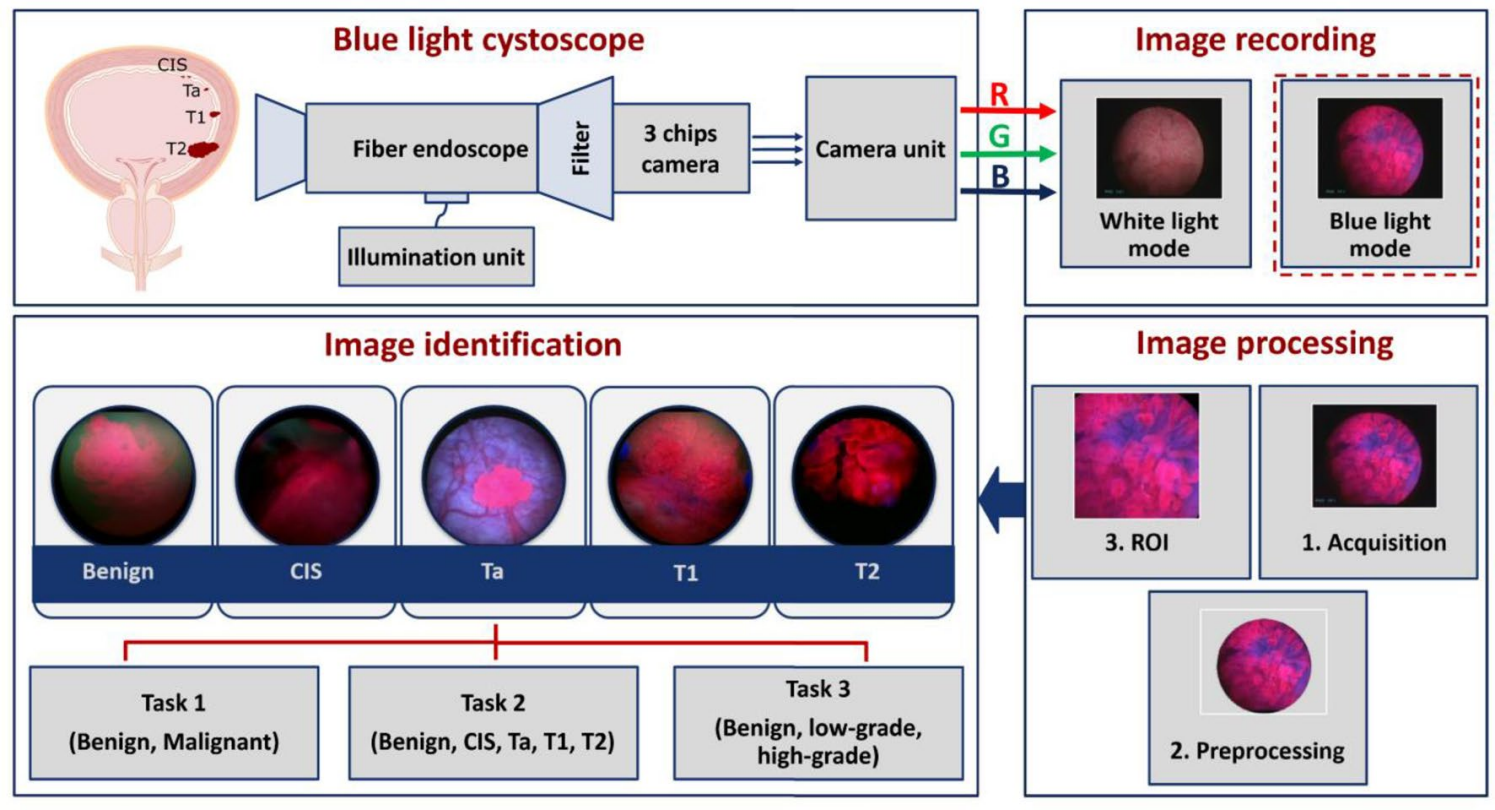
Overview of image acquisition and image processing using the blue light cystoscope.

To improve the identification results of bladder cancer, only the regions of interest (ROI) in the PDD images were included in the data modeling (see Fig. 5). Here, the ROI of an image refers to the image area containing the bladder tissue. This determination of ROIs was performed automatically based on image processing techniques, and it started by enhancing the contrast of the red and blue channels of all PDD images using the contrast limited adaptive histogram equalization algorithm^29^. Thereafter, the tissue area of each image was extracted by fitting a disk in order to remove background areas. Finally, the ROI of an image was acquired as an inscribed square region within the extracted image disk. Applying the previous image preprocessing pipeline returns images of the size of 384 × 384 pixels.

### Transfer learning based on deep convolutional neural networks

**Figure 6:**
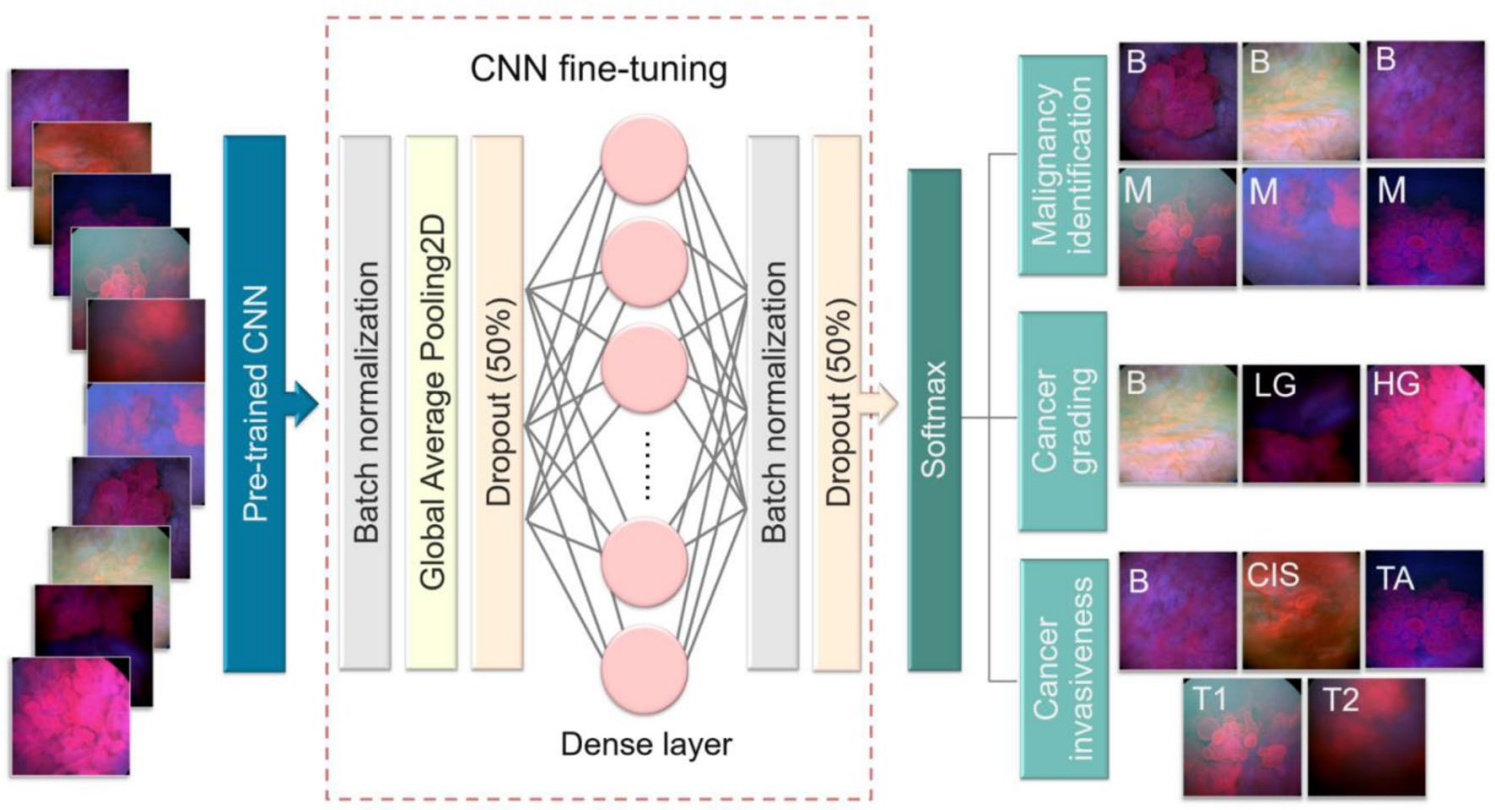
Schematic diagram of the CNN fine-tuning considered for identifying bladder cancer. Each pre-trained CNN was fine-tuned by appending two batch normalization layers, a global average pooling layer, dropout layers with the probability of 50%, a dense layer to improve cancer identification, and Softmax activation layer. The last layer delivers different label probabilities for each input image with respect to each classification task.

The basic idea of transfer learning for deep learning models is to utilize knowledge gained by training a deep neural network on a large and annotated data set to solve another classification task^12,13^. In this contribution, we proposed a transfer learning strategy in which an ensemble of different pre-trained deep CNNs were fine-tuned to improve the classification of BL images. The respective CNNs are the InceptionV3 network^30^, MobileNetV2 network^31^, ResNet50 network^32^ and VGG16 network^33^, and they represent common freely available fully connected CNNs that were pre-trained on the ImageNet dataset. As we mentioned, the identification ability of the previous pre-trained CNNs can be transferred via a fine-tuning approach, which was accomplished by appending additional layers on top of each network. These additional layers are two batch normalization layers, a global average pooling layer, dropout layers with the probability of 50%, and a dense layer to find the best combinations of the already learnt features that improve bladder cancer identification. The last additional layer is a Softmax activation layer, which provides label probabilities for each image with respect to the considered classification task (see Fig. 6 for more details). The parameters of these layer were optimized by an Adam optimizer, which was trained for 100 epochs based on a mini-batch of 5 patches. The optimization hyperparameters were a learning rate of 0.001 and the categorical-cross entropy loss function. Class imbalance was tackled by the SckiKit Learn function *class_weight.compute_class_weight (‘balanced’)*.

### Image augmentation and cross validation

The input image size of all previous CNNs was fixed to be 224 × 224 pixels; therefore, all endoscopic images were first down-sampled to that size. Then, class labels of all down-sampled images were predicted four times based on the four fine-tuned CNNs. These CNN were evaluated using the L10PO-CV as a validation strategy. Therein, we always fixed 10 images (from 10 patients) as test set and 10 images as validation set while the remaining images were utilized to train the considered fine-tuned CNN. The last procedure was repeated 22 times until labels of all images (and patients) were predicted by all fine-tuned CNNs. Because the training set has a maximum size of 196 images per each cross-validation iteration, the BL images utilized to train the CNNs within each iteration were augmented automatically using random rotations by steps of 10° degrees within the range of 0° to 180°. Thereafter, each fine-tuned CNN was trained for 100 epochs based on a mini-batch of 5 patches and using the Adam optimizer with a learning rate of 0.001 to minimize the categorical-cross entropy loss function.

### Data modeling and models evaluation

For interpretation of the cross-validation results, we calculated the confusion matrix and the classification sensitivity and specificity with respect to all tested classification tasks and all fine-tuned CNNs. For ratings of the urologists, confusion matrices were also computed by comparing these ratings with the image ground truth, e.g., the pathological annotation of the biopsied tissue sample. Lastly, the resulting mean sensitivities and mean specificities were calculated for all CNNs results and for both urologists’ results.

All computations in this work were accomplished based on in-house written functions using the programming language Python version 3.7^34^ and the statistical programing language R version 3.6^35^.

## Supplementary Information

Supplementary Information.

## References

[1] Antoni S (2017). Bladder cancer incidence and mortality: A global overview and recent trends. Eur. Urol..

[2] Burger M (2013). Epidemiology and risk factors of urothelial bladder cancer. Eur. Urol..

[3] Cina SJ (2001). Correlation of cystoscopic impression with histologic diagnosis of biopsy specimens of the bladder. Hum. Pathol..

[4] Burger M (2013). Photodynamic diagnosis of non-muscle-invasive bladder cancer with hexaminolevulinate cystoscopy: A meta-analysis of detection and recurrence based on raw data. Eur. Urol..

[5] Rink M (2013). Hexyl aminolevulinate-guided fluorescence cystoscopy in the diagnosis and follow-up of patients with non-muscle-invasive bladder cancer: A critical review of the current literature. Eur. Urol..

[6] Daneshmand S (2018). Blue light cystoscopy for the diagnosis of bladder cancer: Results from the US prospective multicenter registry. Urol. Oncol..

[7] Gravas S (2012). Is there a learning curve for photodynamic diagnosis of bladder cancer with hexaminolevulinate hydrochloride?. Can. J. Urol..

[8] Saba L (2019). The present and future of deep learning in radiology. Eur. J. Radiol..

[9] Srinidhi, C., Ciga, O., & Martel, A. *Deep neural network models for computational histopathology: A survey.* (2019). http://arxiv.org/abs/1912.12378. 10.1016/j.media.2020.101813 PMC7725956 33049577

[10] Kietzmann TC, McClure P, Kriegeskorte N (2019). Deep Neural Networks in Computational Neuroscience.

[11] Cichy RM (2016). Comparison of deep neural networks to spatio-temporal cortical dynamics of human visual object recognition reveals hierarchical correspondence. Sci. Rep..

[12] Shin HC (2016). Deep convolutional neural networks for computer-aided detection: CNN Architectures, dataset characteristics and transfer learning. IEEE Trans. Med. Imaging.

[13] Pradhan P (2020). Deep learning a boon for biophotonics?. J. Biophoton..

[14] Ali N (2019). Automatic label-free detection of breast cancer using nonlinear multimodal imaging and the convolutional neural network ResNet50. Transl. Biophoton..

[15] Aubreville M (2017). Automatic classification of cancerous tissue in laserendomicroscopy images of the oral cavity using deep learning. Sci. Rep..

[16] Wu E (2019). Deep learning approach for assessment of bladder cancer treatment response. Tomography.

[17] Xu H (2019). Using transfer learning on whole slide images to predict tumor mutational burden in bladder cancer patients. BioRxiv.

[18] Tokas T (2018). A 12-year follow-up of ANNA/C-TRUS image-targeted biopsies in patients suspicious for prostate cancer. World J. Urol..

[19] Xue J (2019). Deep learning-based detection and segmentation-assisted management of brain metastases. Neuro Oncol..

[20] Shkolyar E (2019). Augmented bladder tumor detection using deep learning. Eur. Urol..

[21] Gosnell ME (2018). Computer-assisted cystoscopy diagnosis of bladder cancer. Urol. Oncol..

[22] Mari A (2018). Novel endoscopic visualization techniques for bladder cancer detection: A review of the contemporary literature. Curr. Opin. Urol..

[23] Goh AC (2008). Optical coherence tomography as an adjunct to white light cystoscopy for intravesical real-time imaging and staging of bladder cancer. Urology.

[24] Wu J (2019). Optical biopsy of bladder cancer using confocal laser endomicroscopy. Int. Urol. Nephrol..

[25] Soria F (2019). The rational and benefits of the second look transurethral resection of the bladder for T1 high grade bladder cancer. Transl. Androl. Urol..

[26] Nassiri N (2020). Detecting invisible bladder cancers with blue light cystoscopy. Urology.

[27] Schmidbauer J (2009). Fluorescence cystoscopy with high-resolution optical coherence tomography imaging as an adjunct reduces false-positive findings in the diagnosis of urothelial carcinoma of the bladder. Eur. Urol..

[28] Lotan Y (2019). Blue light flexible cystoscopy with hexaminolevulinate in non-muscle-invasive bladder cancer: Review of the clinical evidence and consensus statement on optimal use in the USA: Update 2018. Nat. Rev. Urol..

[29] Reza AM (2004). Realization of the contrast limited adaptive histogram equalization (CLAHE) for real-time image enhancement. J. VLSI Signal Process..

[30] Szegedy, C. *et al*. *Rethinking the inception architecture for computer vision*. In *2016 IEEE Conference on Computer Vision and Pattern Recognition (CVPR)* (2016).

[31] Sandler, M. *et al*. *MobileNetV2: Inverted residuals and linear bottlenecks*. In *2018 IEEE/CVF Conference on Computer Vision and Pattern Recognition* (2018).

[32] He, K. *et al.**Deep residual learning for image recognition*. In *2016 IEEE Conference on Computer Vision and Pattern Recognition (CVPR)* (2016).

[33] Simonyan, K. & Zisserman, A. *Very Deep Convolutional Networks for Large-Scale Image Recognition* (2014). http://arxiv.org/abs/1409.1556.

[34] Van Rossum, G.A.D. & Fred, L. *Python 3 Reference Manual*. (CreateSpace, 2009).

[35] R Core Team, *R: A Language and Environment for Statistical Computing*. (R Foundation for Statistical Computing, 2019).

