# Supplementary material for: Deep learning-based classification of blue light cystoscopy imaging during transurethral resection of bladder tumors
Source: Sci Rep. 2021 Jun 2;11:11629. doi: 10.1038/s41598-021-91081-x (PMC8172542; doi:10.1038/s41598-021-91081-x)
Supplement: Supplementary file 1 — Supplementary Information. [file 41598_2021_91081_MOESM1_ESM.docx]

**Supplementary Materials for:**

**Deep learning-based classification of blue light cystoscopy imaging during transurethral resection of bladder tumors**

Nairveen Ali ^1,2^, Christian Bolenz ^3^, Tilman Todenhöfer ^4^, Arnuf Stenzel ^4^, Peer Deetmar ^5^, Martin Kriegmair ^6^, Thomas Knoll ^7^, Stefan Porubsky ^8^, Arndt Hartmann ^9^, Jürgen Popp ^1,2^, Maximilian C. Kriegmair ^10,*^, Thomas Bocklitz ^1,2,*^

^1^ Institute of Physical Chemistry and Abbe Center of Photonics (IPC), Friedrich-Schiller-University, Jena, Germany

^2^ Leibniz Institute of Photonic Technology (IPHT), Jena, Germany

^3^ Department of Urology, University of Ulm, Ulm, Germany

^4^ Department of Urology, University Hospital Tübingen, Tübingen, Germany

^5^ Pathology Munich-Nord, Munich, Germany

^6^ Urological Hospital Munich-Planegg, Germany

^7^ Department of Urology, Hospital Sindelfingen-Böblingen, University of Tübingen, Sindelfingen, Germany

^8^ Institute of Pathology, University Medical Center of the Johannes Gutenberg University Mainz, Mainz, Germany

^9^ Institute of Pathology, University of Erlangen, Erlangen, Germany

^10^ Department of Urology, University Medical Centre Mannheim, Mannheim, Germany

* Corresponding authors

**Table S1.** The confusion tables of bladder cancer malignancy identification. The results here were obtained by the fine-tuned CNNs based on the cross- validation and by physician ratings.

| Model | True | Prediction | | Sens. | Model | True | Prediction | | Sens. |
| --- | --- | --- | --- | --- | --- | --- | --- | --- | --- |
|  |  | Benign | Malignant |  |  |  | Benign | Malignant |  |
| **IncepctionV3** | Benign | 57 | 17 | 77.03% | **ResNet50** | Benign | 61 | 13 | 82.43% |
|  | Malignant | 7 | 135 | 95.07% |  | Malignant | 12 | 129 | 91.55% |
| **MobileNetV2** | Benign | 65 | 9 | 87.84% | **VGG16** | Benign | 65 | 9 | 87.84% |
|  | Malignant | 6 | 136 | 95.77% |  | Malignant | 9 | 133 | 93.66% |
| **Physician 1** | Benign | 39 | 35 | 52.70% | **Physician 2** | Benign | 41 | 33 | 56.41% |
|  | Malignant | 17 | 125 | 88.03% |  | Malignant | 18 | 124 | 87.32% |

**Table S2.** The prediction results of cancer stage based on the fine-tuned CNNs and physician ratings.

| Model | True | Prediction | | | | | Sens. | Spec. |
| --- | --- | --- | --- | --- | --- | --- | --- | --- |
|  |  | **Benign** | **Ta** | **T1** | **T2** | **CIS** |  |  |
| IncepctionV3 | Benign | 62 | 5 | 3 | 4 | 0 | 83.78% | 94.37% |
|  | Ta | 4 | 91 | 2 | 2 | 1 | 91% | 91.38% |
|  | T1 | 1 | 3 | 10 | 0 | 0 | 71.43% | 97.52% |
|  | T2 | 1 | 0 | 0 | 10 | 0 | 90.91% | 96.10% |
|  | CIS | 2 | 2 | 0 | 2 | 11 | 64.71% | 99.50% |
| MobileNetV2 | Benign | 59 | 7 | 3 | 1 | 4 | 79.72% | 98.59% |
|  | Ta | 1 | 93 | 2 | 2 | 2 | 93% | 92.24% |
|  | T1 | 0 | 0 | 14 | 0 | 0 | 100% | 97.52% |
|  | T2 | 0 | 1 | 0 | 10 | 0 | 90.91% | 97.56% |
|  | CIS | 1 | 1 | 0 | 2 | 13 | 76.47% | 96.98% |
| ResNet50 | Benign | 58 | 13 | 0 | 2 | 1 | 78.38% | 97.18% |
|  | Ta | 3 | 92 | 2 | 3 | 0 | 92% | 81.90% |
|  | T1 | 0 | 4 | 10 | 0 | 0 | 71.43% | 98.51% |
|  | T2 | 0 | 0 | 0 | 11 | 0 | 100% | 96.58% |
|  | CIS | 1 | 4 | 1 | 2 | 9 | 52.94% | 99.50% |
| VGG16 | Benign | 60 | 5 | 0 | 6 | 3 | 81.08% | 95.77% |
|  | Ta | 2 | 91 | 2 | 1 | 4 | 91% | 91.37% |
|  | T1 | 1 | 2 | 9 | 1 | 1 | 64.29% | 99.01% |
|  | T2 | 0 | 2 | 0 | 8 | 1 | 72.72% | 95.61% |
|  | CIS | 3 | 1 | 0 | 1 | 12 | 70.59% | 95.48% |
| Physician 1 | Benign | 39 | 25 | 2 | 1 | 7 | 52.70% | 88.03% |
|  | Ta | 6 | 84 | 7 | 0 | 3 | 84.00% | 61.21% |
|  | T1 | 3 | 6 | 2 | 0 | 1 | 16.67% | 93.63% |
|  | T2 | 1 | 9 | 3 | 0 | 0 | 0% | 99.50% |
|  | CIS | 7 | 5 | 1 | 0 | 4 | 23.53% | 94.47% |
| Physician 2 | Benign | 41 | 21 | 2 | 2 | 8 | 55.41% | 87.32% |
|  | Ta | 7 | 82 | 7 | 0 | 4 | 82.00% | 64.66% |
|  | T1 | 3 | 6 | 3 | 0 | 0 | 25.00% | 93.62% |
|  | T2 | 1 | 9 | 3 | 0 | 0 | 0% | 99.01% |
|  | CIS | 7 | 5 | 1 | 0 | 4 | 23.53% | 93.96% |

**Table S3.** Confusion matrices obtained by the deep learning models and physician ratings.

| Model | True | Prediction | | | Sens. | Spec. |
| --- | --- | --- | --- | --- | --- | --- |
|  |  | Benign | Low-grade | High-grade |  |  |
| IncepctionV3 | Benign | 57 | 7 | 10 | 77.03% | 85.92% |
|  | Low-grade | 8 | 57 | 8 | 78.08% | 93.01% |
|  | High-grade | 12 | 3 | 54 | 78.26% | 87.76% |
| MobileNetV2 | Benign | 63 | 5 | 6 | 85.14% | 95.07% |
|  | Low-grade | 4 | 67 | 2 | 91.78% | 93.01% |
|  | High-grade | 3 | 5 | 61 | 88.44% | 94.56% |
| ResNet50 | Benign | 71 | 2 | 1 | 95.95% | 93.66% |
|  | Low-grade | 4 | 66 | 4 | 90.41% | 97.20% |
|  | High-grade | 5 | 2 | 62 | 89.86% | 97.27% |
| VGG16 | Benign | 62 | 7 | 5 | 83.80% | 89.43% |
|  | Low-grade | 7 | 61 | 5 | 83.56% | 93.01% |
|  | High-grade | 8 | 3 | 58 | 84.06% | 93.20% |
| Physician 1 | Benign | 39 | 23 | 12 | 52.70% | 88.02% |
|  | Low-grade | 4 | 58 | 11 | 79.45% | 58.74% |
|  | High-grade | 13 | 36 | 20 | 28.99% | 84.35% |
| Physician 2 | Benign | 41 | 19 | 14 | 56.40% | 87.32% |
|  | Low-grade | 3 | 55 | 15 | 75.34% | 65.73% |
|  | High-grade | 15 | 30 | 24 | 34.78% | 80.27% |

**Table S4.** A comparison between binary CNN models and multiclass CNN models in image identification of benign and carcinoma in situ (CIS) bladder lesions.

| Binary model | | | | Multiclass model | | | |
| --- | --- | --- | --- | --- | --- | --- | --- |
| **Model** | **Class** | **Sens.** | **M. Sens.** | **Model** | **True** | **Sens.** | **M. Sens.** |
| **IncepctionV3** | Benign | 90.78% | 57.03% | **IncepctionV3** | Benign | 83.78% | 74.35% |
|  | CIS | 23.52% |  |  | CIS | 64.71% |  |
| **MobileNetV2** | Benign | 56.75% | 60.73% | **MobileNetV2** | Benign | 79.72% | 78.10% |
|  | CIS | 64.70% |  |  | CIS | 76.47% |  |
| **ResNet50** | Benign | 89.19% | 56.35% | **ResNet50** | Benign | 78.38% | 65.66% |
|  | CIS | 23.53% |  |  | CIS | 52.94% |  |
| **VGG16** | Benign | 97.29% | 51.59% | **VGG16** | Benign | 81.08% | 75.84% |
|  | CIS | 05.88% |  |  | CIS | 70.59% |  |
